# OCTN1 mediates acetylcholine transport in the A549 lung cancer cells: possible pathophysiological implications

**DOI:** 10.3389/fmolb.2024.1512530

**Published:** 2024-12-09

**Authors:** Lorena Pochini, Giusi Elisabetta Tedesco, Tiziano Mazza, Mariafrancesca Scalise, Cesare Indiveri

**Affiliations:** ^1^ Laboratory of Biochemistry, Molecular Biotechnology and Molecular Biology, Department DiBEST (Biologia, Ecologia, Scienze Della Terra), University of Calabria, Arcavacata di Rende, Italy; ^2^ Institute of Biomembranes, Bioenergetics and Molecular Biotechnologies (IBIOM), National Research Council (CNR), Bari, Italy

**Keywords:** SLC, lung cancer, non-neuronal cholinergic system, Octn1, drug discovery

## Abstract

A role for acetylcholine in cell proliferation, epithelial mesenchymal transition and invasion has been well assessed and related to the presence of the non-neuronal cholinergic system in lung cancer. For the operation of this non-neuronal system, acetylcholine should be released by a transporter mediated non-quantal process. OCTN1 is one of the transporters able to catalyse acetylcholine efflux *in vitro* and *ex vivo*. Using the A549 cell line as a lung cancer model, it has been found that these cells express OCTN1 at a higher level with respect to other cancer cells. The transport capacity of OCTN1 extracted from A549 and reconstituted into proteoliposomes reflects the protein expression profile. The properties of the acetylcholine transport mediated by OCTN1 of A549 in terms of specificity to ligands and ability to catalyse efflux of acetylcholine correspond to those previously described for the same transporter in other cells or to those of the human recombinant protein. OCTN1 is the major player in acetylcholine release in A549 and, therefore, may represent a target for inhibitors able to block the acetylcholine action in this type of aggressive tumors.

## 1 Introduction

Besides its universally known role as a neurotransmitter, acetylcholine (ACh) is among the factors promoting cancer, as well (Parnell et al., 2012; Zoli et al., 2018), having a role in cell proliferation, Epithelial Mesenchymal Transition (EMT) and invasion (Friedman et al., 2019; Xu et al., 2015; Yang et al., 2014; Chen et al., 2019; Aronowitz et al., 2022; Wu et al., 2022; Niu and Lu, 2014). The function of ACh in human cancers is related to the non-neuronal cholinergic system (NNCS) (Friedman et al., 2019; Yang et al., 2014; Aronowitz et al., 2022; Wu et al., 2022; Saracino et al., 2013) that is present in many tissues including the airway (Wessler et al., 1998). NNCS expression has been demonstrated in both bronchial and alveolar lung epithelia; lung macrophages, that are among the players of NNCS, cannot be reached by the ACh produced by the canonical neuronal cholinergic system (Friedman et al., 2019; Saracino et al., 2013; Kummer et al., 2008; Song and Spindel, 2008; Islas-Weinstein et al., 2020). A role of the non-neuronal ACh has also been established in the control of inflammation that has a strict relationship with lung cancer (Saracino et al., 2013; Gomes et al., 2014; Tan et al., 2021; Mashimo et al., 2021; Banzato et al., 2021; Halder and Lal, 2021; Cox et al., 2020). ACh’s role in regulating immune-mediated inflammation during acute viral infection has been investigated as well and, in this context, cholinergic lymphocytes were found in direct physical contact with activated macrophages throughout the lung (Horkowitz et al., 2020). The ACh synthesis and action are performed in the NNCS by the same pathway occurring in neurons, that is the Choline Acetyl Transferase, the Acetylcholine Receptors and the Esterase which are present in several types of non-neuronal cells including endothelial cells, epithelia, as well as inflammatory and immune cells, such as macrophagic cells, neutrophils, and lymphocytes (Reichrath et al., 2016; Wessler and Kirkpatrick, 2008). But, unlike neurons, in the NNCS, ACh is released by a non-quantal process, that does not involve vesicular accumulation and release. Hence, plasma membrane transporters play a crucial role in NNCS, mediating a slow release of ACh, thus representing one of the main differences between the neuronal vs. the non-neuronal cholinergic systems. Some transporters of the SLC22 family have been shown to mediate ACh transport, among which is the SLC22A4, also known as Organic Cation Transporter Novel 1 (OCTN1). It is acknowledged as responsible for non-quantal ACh release in tissues such as lungs (Kummer and Krasteva-Christ, 2014) where it is expressed both in epithelia and in immune cells (Berg et al., 2018). The OCTN1-mediated release of ACh has been described in HeLa cells (Pochini et al., 2015; Pochini et al., 2022). The ability of OCTN1 to transport ACh has also been characterized in the *in vitro* system of proteoliposomes (Pochini et al., 2012a; Pochini et al., 2012b) and in primary mesothelial cells (Pochini et al., 2016). According to its action mechanism involving cell release, ACh has been suggested to be transported preferentially by an efflux mode; indeed, while extraliposomal or extracellular Na^+^ inhibits the OCTN1-mediated uptake of ACh, the ACh efflux is not influenced by the external Na^+^. Moreover, internal K^+^ does not affect the efflux of ACh (Pochini et al., 2015; Pochini et al., 2012b). Based on these findings, ACh efflux can occur under physiological conditions, i.e., high levels of external sodium and internal potassium. The described features suggest that OCTN1 could be exploited to target the NNCS in lung cancer, which is listed among the most aggressive human tumors, implying difficulties in pharmacological treatments due to recurrent resistance to anticancer drugs (Ashrafi et al., 2022). For a long time, the cholinergic receptors, playing a crucial role in promoting tumor growth and progression in lung cancers, have been considered as potentially targetable (Song and Spindel, 2008; Russo et al., 2014). Nevertheless, the use of receptor antagonists leads to side effects, due to their role in modulating many vital functions (Chen et al., 2019). From a very new perspective, targeting the transporter responsible of cell release of Ach in tumors might be a valuable alternative. Indeed, membrane transporters have been suggested in many cases as more reliable pharmacological targets with respect to intracellular enzymes (Wang et al., 2020; Galetin et al., 2024). Indeed, inhibitors of the serotonin transporter were successfully used for a safer treatment of depression (Singh et al., 2023) and inhibitors of the glucose transporters have been adopted for the treatment of type 2 diabetes (Hiraizumi et al., 2024). Very recently, a transporter inhibition strategy has been under clinical trial for the treatment of pancreatic cancer by JPH203, which is a LAT1 transporter inhibitor (Nishikubo et al., 2022; Brunocilla et al., 2023). Based on the reported dependence of lung cancers on ACh signalling and on the capacity of OCTN1 in exporting ACh, we have investigated the expression and the function of OCTN1 in the widely used cell model of lung cancer, A549 (Wu et al., 2022; Shahzadi et al., 2023). This working hypothesis is also supported by the previous finding that among roughly 30 human drug-transporters, OCTN1 proved to be the most expressed in human lung tissue, thus representing the best target transporter for drug design in lung (Sakamoto et al., 2013).

## 2 Materials and methods

### 2.1 Materials

Sephadex G-75, acetylcholine chloride, Amberlite XAD-4, egg yolk phospholipids (3-sn-phosphatidylcholine from egg yolk), cocktail of protease inhibitors and Triton X-100 were purchased from Merck. [Acetyl-^3^H] acetylcholine iodide was from Perkin–Elmer. HEK293, HepG2 and HeLa cell lines were kindly provided by Dr. Massimo Tommasino (IARC/CIRC WHO, Lyon France); A549, HCT-15, MCF-7 cell lines were kindly provided by Dr. Daniela Gaglio (CNR- IBFM, Milan). Tissue culture media and foetal bovine serum were from Life Technologies. Goat anti-Rabbit IgG (H + L) Secondary Antibody, HRP was from Invitrogen. Anti-SLC22A4 antibody was from ABCAM. Anti-alpha tubulin antibody, mouse monoclonal and Goat Anti-Mouse IgG Antibody, HRP conjugate were from Merck. All the other reagents were of analytical grade.

### 2.2 Reconstitution in proteoliposomes of the hOCTN1 transporter extracted from cancer cells

From each cell line, the transporter was solubilized by treating a cell pellet, from 10 cm^2^ plates up to 90% confluence with 100 μL 3% TX-100 in 20 mM Tris/HCl pH 7.5, 2.5 mM Sodium pyrophosphate, 1 mM β-glycerophosphate, 1 mM Na_3_VO_4_ and 1 μg/mL leupeptin, as previously described (Pochini et al., 2015). After incubation on ice for 30 min the lysate was centrifuged at 12,000 g for 15 min at 4°C. The supernatant (containing cell membrane extract) was quantified using Lowry Folin assay and used for the reconstitution. The mixture for reconstitution contained: 20–40 μL of the cell supernatant (200 μg proteins), 80 μL of 10% TritonX-100, 120 μL of 10% egg yolk phospholipids in the form of sonicated liposomes prepared as described previously (Pochini et al., 2011), 16 mM ATP and 5 mM Tris/HCl (pH 7.5) in a final volume of 700 μL. After vortex-mixing, this mixture was incubated with 0.5 g of Amberlite XAD-4 under rotatory stirring (1,300 rev/min) at 23°C for 45 min.

### 2.3 Cell culture

HEK293 and HeLa cell lines were maintained in Dulbecco’s Modified Eagle Medium (DMEM) supplemented with 10% (v/v) Fetal Bovine Serum (FBS), 1 mM glutamine, 1 mM sodium pyruvate and Pen/strep as antibiotics. HCT-15 cell line was maintained in RPMI (with glucose) supplemented with 10% (v/v) Fetal Bovine Serum (FBS), 2 mM glutamine, and Pen/strep as antibiotics. A549, HepG2 and MCF-7 cell lines were maintained in Dulbecco’s Modified Eagle Medium (DMEM) supplemented with 10% (v/v) Fetal Bovine Serum (FBS), 4 mM glutamine, 1 mM sodium pyruvate and Pen/strep as antibiotics. Cells were grown on 10 cm^2^ plates at 37°C in a humidified incubator and a 5% CO_2_ atmosphere.

### 2.4 Transfection of siRNA in A549 cells

A549 cells were seeded in a 6-well plate 24 h prior transfection and cultured using standard conditions until they reached 80% confluence. On the day of transfection, Lipofectamine RNAiMAX reagent (from Ambion by Life Technologies) was used according to manufacturer’s instructions. In brief, desired amount of siRNA scramble or OCTN1-targeting (Mission esiRNA from Merck Life Science) were diluted with Opti-MEM medium (from Gibco by Life Technologies); in parallel, lipofectamine was diluted with the same medium. Then, diluted siRNA was added to diluted Lipofectamine in a 1:1 ratio. The mixture was incubated for 5 min at room temperature. After the incubation, siRNA-lipid complexes were added to seeded A549 cells. After 72 h of transfection incubated at 37°C in a 5% CO_2_ incubator, cells were used in downstream applications.

### 2.5 RNA extraction and RT-PCR

Total cellular RNA was extracted from A549 pellet, deriving from a confluent 75 cm^2^ flask, using the pureLink RNA mini kit (Invitrogen). Then, RevertAid First Strand cDNA Synthesis Kit (Invitrogen) was used to synthesize cDNA with random hexa primers starting from 1 µg of total RNA. Reverse transcriptase PCR (RT-PCR) analyses were carried out using the following primers: FW 5′CCTGCCCAGGCGTTATATCAT 3′ and Rev 3′CTGCTGAGCTCTACCCAACC5’ (for OCTN1), FW 5′TTTTGTGAGAGCCGTGACTG3′ and Rev 3′CGGTTGCTCATCAGGTAGGT (for ChAT), FW 5′CTGGGAGTGGGTGGAGGC3′ and Rev 3′ TCAACTGGTCTCAAGTCAGTG 5’ (for Actin).

### 2.6 Transport measurements in proteoliposomes

Uptake and efflux assays in proteoliposomes were performed as previously described (Pochini et al., 2012b). In brief, 550 μL of proteoliposomes were subjected to size-exclusion chromatography onto a Sephadex G-75 column (0.7 cm diameter × 15 cm height) pre-equilibrated with 5 mM Tris/HCl (pH 7.5). Then, samples of 100 μL each of the collected proteoliposomes (600 μL) were used for transport measurements. In the case of uptake assays, transport was started by adding 0.1 mM [^3^H]ACh to proteoliposomes and stopped according to the stop inhibitor method. In the case of efflux measurements, aliquots of proteoliposomes were incubated with 0.1 mM external [^3^H]ACh. After 90 min, corresponding to the optimal intraliposomal [^3^H]ACh accumulation the proteoliposomes were passed again through a Sephadex G-75 column and the time course of [^3^H]ACh efflux was then measured as described for the uptake procedure. Finally, the radioactivity taken up (or remained inside in the case of efflux) was counted after passing each 100 μL sample through a Sephadex G-75 column (0.6 cm diameter × 8 cm height) to separate the external from the internal radioactivity. The experimental values were analysed using a first order rate equation or a single exponential decay equation, in the case of uptake or efflux, respectively. The Grafit (version 5.0.13) software was used for calculations.

### 2.7 Other methods

Protein amount was measured using the ChemiDoc imaging system equipped with Quantity One software (Bio-Rad Laboratories). Immunoblotting analysis was performed on cell extracts obtained as described in Section 2.2 using an antiserum dilution of 1:1000 for all the used primary antibodies prepared in 3% BSA and incubated overnight under shaking at 4°C. Then, 1:5000 secondary antibody (anti-rabbit) was prepared in 1% BSA and incubated 1 h at room temperature under shaking. The reaction was detected by Electro Chemi Luminescence (ECL) assay using the ChemiDoc imaging system equipped with Image Lab software (Bio-Rad Laboratories).

### 2.8 Equations

First order rate equation: A_t_ = A_∞_ (1- e^-kt^) used for uptake assay, where At and A∞ represent the nanomoles of substrate taken up at time t and at infinite time, respectively. Initial transport rate is evaluated as the product of the first-order rate constant, k, and the nanomoles of substrate taken up at the equilibrium.

Single exponential decay equation: y = A_0_ e^-kt^ used for efflux assay where y and A are the internal radioactivity (cpm) at time t and time zero, respectively. The rate constant k is derived from the decrease in the radioactivity inside the liposomes at various times until equilibrium.

### 2.9 Statistical analysis

Results are expressed as means ± SD and the number of replicates is indicated in figure legends. Comparisons between two groups were performed with the two-tailed Student’s unpaired *t*-test for p < 0.05 (*) and p <0.01 (**) as specified in figure legends. For multiple comparisons, ANOVA one way test was employed for *p < 0.05, **p < 0.01, ***p < 0.005 as specified in figure legends.

## 3 Results

### 3.1 OCTN1 expression in cancer cell lines

**FIGURE 1:**
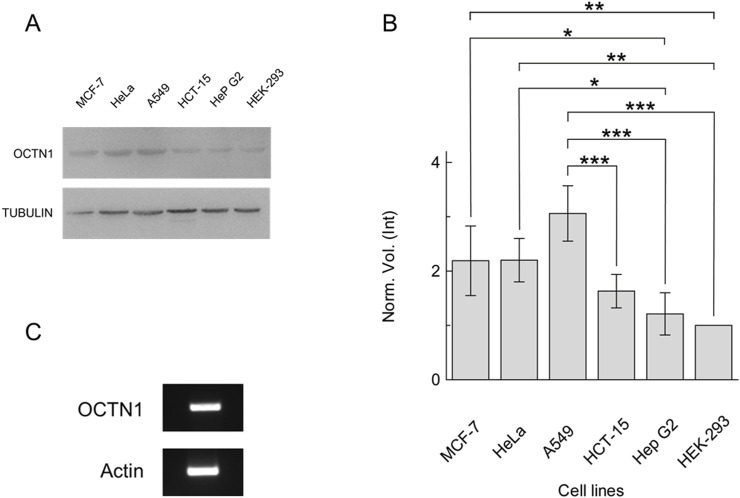
Expression of OCTN1 in cancer cell lines. **(A)** Cell extracts obtained as described in Section 2.2 were loaded on SDS-PAGE and blotted; OCTN1 or tubulin (loading control) was immunodetected by anti-OCTN1 or anti-tubulin, respectively. Representative blots are shown. **(B)** The histogram represents scanning densitometry (±S.D.) of four similar immunoblots reported as the Norm. Vol. (Int) compared to OCTN1 from HEK-293 cell line. Significantly different as estimated by One Way ANOVA followed by Tukey test for *p < 0.05, **p < 0.01, ***p < 0.005. **(C)** OCTN1 mRNA identification in A549, RT-PCR of OCTN1 and control (Actin) were performed as described in Section 2.5.

The expression of OCTN1 has been investigated in the non-small cell lung cancer cell line A549 in comparison with other cell lines (Figures 1A, B), prototypes of aggressive cancers, most of which are also known to rely on non-neuronal ACh signalling (Parnell et al., 2012; Friedman et al., 2019; Aronowitz et al., 2022; Zhang et al., 2020); HEK293 was employed as control due to the relatively low level of OCTN1 expression (Tamai et al., 1997; Drenberg et al., 2017; Tamai et al., 2000). A protein band appeared in all cell extracts with different intensities (Figure 1A). The quantitative analysis, using Alpha-tubulin as a loading control, revealed that A549 expressed OCTN1 at a significantly higher level compared to the other cell lines (Figures 1A, B). The expression of OCTN1 mRNA was confirmed in A549 by RT-PCR, as well (Figure 1C). To confirm the existence of the NNCS in lung cancer the Choline Acetyltransferase was identified by RT-PCR (Supplementary Figure 1).

### 3.2 OCTN1 functional identification

**FIGURE 2:**
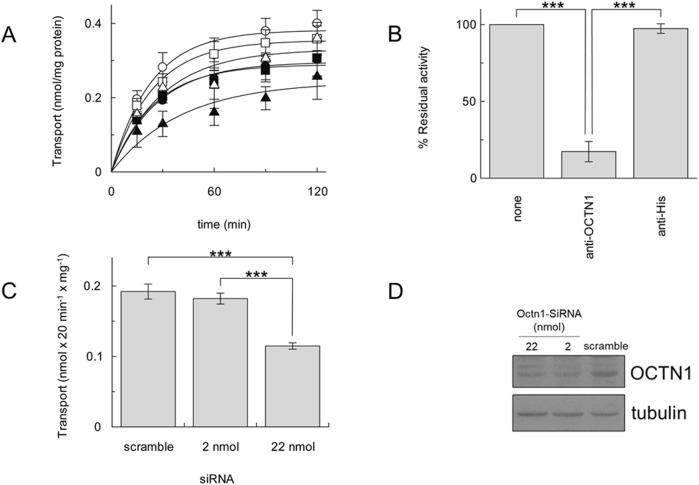
[^3^H]ACh transport by cancer cell line OCTN1. **(A)**, time course of [^3^H]ACh in proteoliposomes reconstituted with cancer cell extracts of A549 (○), MCF-7 (●), HeLa (□), Hep G2 (■), HCT-15 (Δ), HEK-293 (▲). **(B)**, Uptake (30 min) of 0.1 mM [^3^H]ACh into proteoliposomes reconstituted with A549 cell extract in the presence of anti-OCTN1 or anti-His reported as residual activity with respect to the 100% control (absence of an antibody). **(C)**, Uptake (20 min) of 0.1 mM [^3^H]Ach in proteoliposomes reconstituted with protein extract from A549 cells transfected with siRNA scramble (control) or the indicated amount (pmol) of OCTN1-targeting siRNA. All data are means ± S.D. of three **(B)** or four **(C)** experiments. Significantly different as estimated by One Way ANOVA followed by Tukey test for *p < 0.05, **p < 0.01, ***p < 0.005. **(D)**, A549 cell extracts obtained as described in Section 2.2 were loaded on SDS-PAGE and blotted; OCTN1 or tubulin (loading control) was immunodetected by anti-OCTN1 or anti-tubulin, respectively. Indicated nmol of SiRNA targeting Octn1 were used with respect to control (scramble SiRNA). Image is a representative blot of two experiments.

As a reliable proof of OCTN1-mediated ACh transport, the time course of [^3^H]ACh transport was measured into proteoliposomes reconstituted with the different cancer cell extracts, as previously described (Pochini et al., 2015; Pochini et al., 2016). In agreement with the Western blot, A549 showed a higher transport activity followed by HeLa, HCT-15, MCF-7, HepG2 and HEK-293 (Figure 2A). To prove the specificity of the OCTN1-mediated ACh transport, proteoliposomes reconstituted with the A549 cell extract, containing the cell membrane proteins, were incubated with the anti-OCTN1 antibody; the uptake of [^3^H]ACh decreased by 83% (Figure 2B). As a control, the uptake of [^3^H]ACh was measured after incubation with an anti-His antibody that was previously shown to inhibit the recombinant human OCTN1 harbouring a 6 His tag (Pochini et al., 2015). The anti-His did not influence the activity of the A549 transporter confirming that the measured transport was specifically attributable to the native OCTN1 expressed by A549 (Figure 2B). This data also indicates that most, if not all, ACh transport capacity of A549 is performed by OCTN1. A little residual contribution to the transport of Ach may be due to other transporters known to mediate Ach transport, such as OCTs. However, in agreement with our data, expression of OCTN1 in lung is reported to be higher (Kummer and Krasteva-Christ, 2014; Sakamoto et al., 2013) than OCTs and, in spite of some contradictory data reported for OCTs, the expression of OCTN1 in lung is unequivocally described (Endter et al., 2009; Courcot et al., 2012; Salomon et al., 2012). To further confirm that the transport of [^3^H]ACh was mediated by OCTN1, A549 cell extract derived by transfection with OCTN1-targeting siRNA was used for preparing proteoliposomes; a significant difference was observed in the uptake of [^3^H]ACh with respect to A549 cell extract derived from cells transfected with siRNA scramble (Figures 2C, D).

### 3.3 OCTN1 functional characterization

**FIGURE 3:**
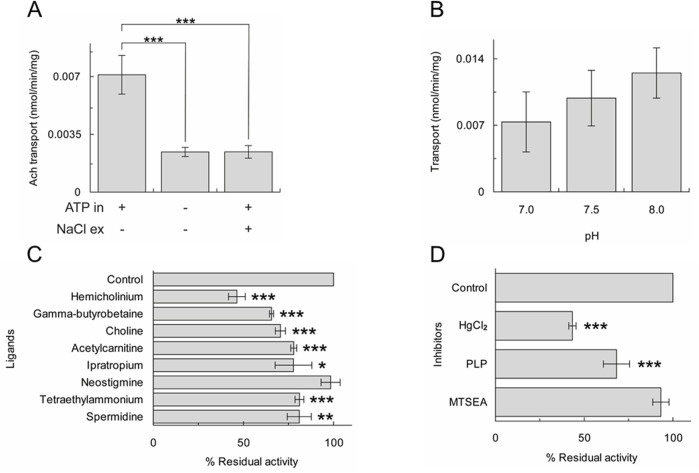
Characterization of [^3^H]ACh transport of A549 OCTN1. Uptake (20 min) of 0.1 mM [^3^H]ACh into proteoliposomes reconstituted with A549 cell extract. Activity measured in the absence or the presence of internal ATP and/or external NaCl **(A)** or at different pH **(B)**. The results are means ± S.D. of three experiments, significantly different as estimated by One Way ANOVA followed by Tukey test for *p < 0.05, **p < 0.01, ***p < 0.005. **(C)** and **(D)** percent residual activity measured in the presence of added ligands **(C)** or inhibitors **(D)**. The results are means ± S.D. of three experiments, significantly different from the control as estimated by Student's t-test for *p < 0.05, **p < 0.01, ***p < 0.005.

Previous studies demonstrated that ATP present in the intracellular (Tamai et al., 1997) or the intraliposomal (Pochini et al., 2012a; Pochini et al., 2011) compartment strongly stimulates the OCTN1-mediated transport; whereas, sodium present in the extracellular (Pochini et al., 2015) or in the extraliposomal (Pochini et al., 2012b) compartment strongly inhibits the uptake. As shown in Figure 3A, [^3^H]ACh uptake in proteoliposomes strongly decreased in the absence of internal ATP or the presence of external sodium. The pH dependence was also investigated; in agreement with previous data obtained in intact cells or proteoliposomes (Pochini et al., 2011; Tamai et al., 1997), transport activity increased by increasing pH from pH 7.0 to pH 8.0, even though with low significance (Figure 3B).

Another specific feature of OCTN1 is its inhibition by choline, TEA, hemicholinium, an inhibitor of the high-affinity uptake system for choline, ipratropium, an ACh receptor antagonist and at a lower extent, by some carnitine analogues and by polyamines that were reported as substrates (Pochini et al., 2015; Pochini et al., 2022). The [^3^H]ACh uptake measured in proteoliposomes reconstituted with the A549 cell extract was significantly inhibited by hemicholinium, γ-butyrobetaine, choline, acetylcarnitine, ipratropium, TEA and spermidine whereas neostigmine had no effect (Figure 3C). The behaviour towards these compounds is similar to that previously described employing the recombinant OCTN1 or the protein extracted from HeLa or mesothelial cells (Pochini et al., 2015; Pochini et al., 2016). The effect of chemical agents that react with sulfhydryl groups (SH reagents) such as 2-aminoethyl methanethiosulfonate (MTSEA) or HgCl_2_ and the Lys reagent pyridoxal phosphate (PLP) was tested on the uptake (Figure 3D). Among these, HgCl_2_ was found to strongly inhibit OCTN1 according to previous findings on the recombinant OCTN1 (Pochini et al., 2011).

**FIGURE 4:**
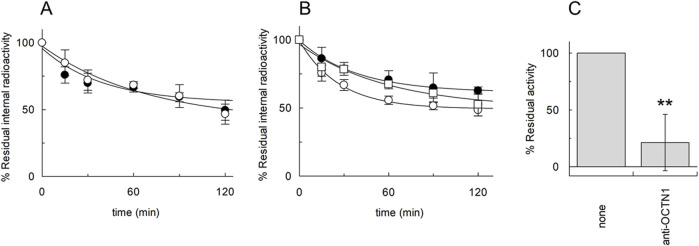
[^3^H]ACh efflux mediated by A549 OCTN1. Efflux of 0.1 mM [^3^H]ACh from proteoliposomes reconstituted with A549 protein extract was measured in the absence (○) or in the presence (●) of 50 mM external NaCl **(A)**; in the presence of 50 mM internal KCl and 50 mM external NaCl (○) or in the presence of external 100 mM sucrose and the presence of 50 mM internal KCl (●) or 50 mM internal NaCl (□) **(B)**. In **(A)** and **(B)** Residual internal radioactivity at the indicated times with respect to time zero was measured. **(C)** Efflux of 0.1 mM [^3^H]Ach in 15 min from proteoliposomes reconstituted with protein extract from A549 cells in the presence or absence of anti-OCTN1 added during reconstitution has been measured. Residual activity with respect to the 100% control (absence of an antibody) is indicated. The values are means ± S.D. from three independent experiments significantly different from the control as estimated by Student’s t-test for **p < 0.01.

As stated in the introduction, the efflux of ACh should be the physiological transport mode of OCTN1 to allow the export of ACh from cells. Therefore, efflux experiments were performed in the presence of external sodium (Figure 4A). Interestingly, [^3^H]ACh efflux was not inhibited by sodium, according to the OCTN1-mediated transport. The effect of internal potassium was also investigated in the absence or the presence of external sodium (Figure 4B). The efflux increased under conditions mimicking the physiological environment, that is with internal potassium and external sodium. To further confirm the OCTN1-mediated [^3^H]ACh efflux, transport has been measured from proteoliposomes reconstituted in the presence of the OCTN1-specific antibody (Figure 4C).

## 4 Discussion

Finding biochemical pathways used by cancer cells for proliferation and invasion is crucial for identifying novel targets for therapy, particularly in those cancers characterized by poor prognosis. In this frame, membrane transporters are considered hot targets for pharmacological intervention since their activity is essential for the completion of specific metabolic and signalling pathways (Galetin et al., 2024). In many tumors, transporter expression is altered with respect to the normal tissues. Moreover, plasma membrane transporters can be considered favorable targets for drugs due to their facing towards the extracellular environment. A relevant role for the NNCS in lung cancer development and progression exists, due to the autocrine and paracrine effects of ACh. In agreement, in A549 we have found higher OCTN1 expression with respect HEK293 cells that do not derive from tumors. Some mRNA-Seq data correlate with OCTN1 expression in lung cancer at a level somewhat higher than in normal lung tissue both in *Mus musculus* (Expression Atlas, https://tinyurl.com/mm5743cr (Choi et al., 2015) and humans (The Human Protein Atlas, https://tinyurl.com/2yhk7x7h).

We here show that the main player in mediating ACh flux in lung cancer cell line A549 is the plasma membrane transporter OCTN1. These results were achieved by employing an experimental strategy based on the reconstitution in proteoliposomes of the cell extracts. The proteoliposome model is suitable for obtaining information on the cell function because this system well mimics the natural membrane environment since the transporter is inserted in the artificial membrane with the same orientation as the native one (Pochini et al., 2011). That the measured transport is specifically mediated by OCTN1 has been also confirmed by the profile of inhibition by some ACh or organic cation analogues that is similar to that previously described for the recombinant OCTN1 (Pochini et al., 2012a; Pochini et al., 2011) (Figure 3C). In addition, the absence of inhibition by neostigmine, the inhibitor of choline acetyl transferase, differentiates the structure/function relationships of OCTN1 from those of the enzyme. The most striking evidence linking OCTN1 transport function to the NNCS in lung cancer is its ability to mediate ACh efflux, which was stimulated when measured in physiological conditions (internal potassium and external sodium) (Kummer and Krasteva-Christ, 2014). OCTN1 in A549 has a similar function to that previously described in HeLa cells (Pochini et al., 2015) indicating that the role of this transporter in releasing ACh might be a hallmark of epithelial cancers. The association of OCTN1 polymorphisms with chronic inflammatory diseases (Pochini et al., 2012a) correlates well with the activity of this transporter in acetylcholine release described *in vitro* using both the recombinant protein (Pochini et al., 2012a), the protein extracted from the membrane of several cancer cell lines, as described in this work, or intact cells (Pochini et al., 2015). Based on our results, OCTN1 could be hypothesized as a novel target for drugs that may impair the NNCS in cancers in which this system is important for proliferation and EMT (Wu et al., 2022; Sales et al., 2019; Zhao et al., 2015). The previous finding that the knockout of OCTN1 in mice does not show a phenotype lets us to also hypothesize that targeting OCTN1 may have relatively low side effects even though the mouse model does not always represent the appropriate *in vivo* model for humans (Pochini et al., 2022). Besides the aforementioned association of OCTN1 polymorphisms with inflammatory diseases (Pochini et al., 2024a), no severe diseases have been described in humans as the consequence of OCTN1 polymorphisms. These observations are again in favor of the expectance of relatively low side effects upon OCTN1 inhibition also in humans. Of course, biological assays on cell or animal models and the use of specific inhibitors will be required to finally demonstrate the role of OCTN1 as a pharmacological target in some human cancers. The resolution of the 3D structure of transporters of the same SLC22 family such as OCT1, 2 and 3 is fundamental for obtaining an accurate structural model of OCTN1 which represents the basis for virtual high throughput screening of ligands (Pochini et al., 2024b). A very recent large *in silico* screening allowed us to describe the binding properties of the transporter and to identify many interactors (Ben Mariem et al., 2024). Additional studies will be further conducted to validate the inhibition property of the identified ligands and, in particular, the ability to inhibit the Ach release activity of OCTN1. Taken together, our results may open new possibilities for improving the chemotherapy response to lung cancer.

## Data Availability

The raw data supporting the conclusions of this article will be made available by the authors, without undue reservation.

## Supplementary material

SUPPLEMENTARY FIGURE 1 Identification of Choline Acetyltransferase in A549 by RT-PCR. RT-PCR of Choline Acetyltranferase (ChAT) and control (Actin) were performed as described in Materials and Methods.
