# Supplementary material for: OCTN1 mediates acetylcholine transport in the A549 lung cancer cells: possible pathophysiological implications
Source: Front Mol Biosci. 2024 Dec 9;11:1512530. doi: 10.3389/fmolb.2024.1512530 (PMC11666908; doi:10.3389/fmolb.2024.1512530)
Supplement: Supplementary file 1 [file DataSheet1.pdf]

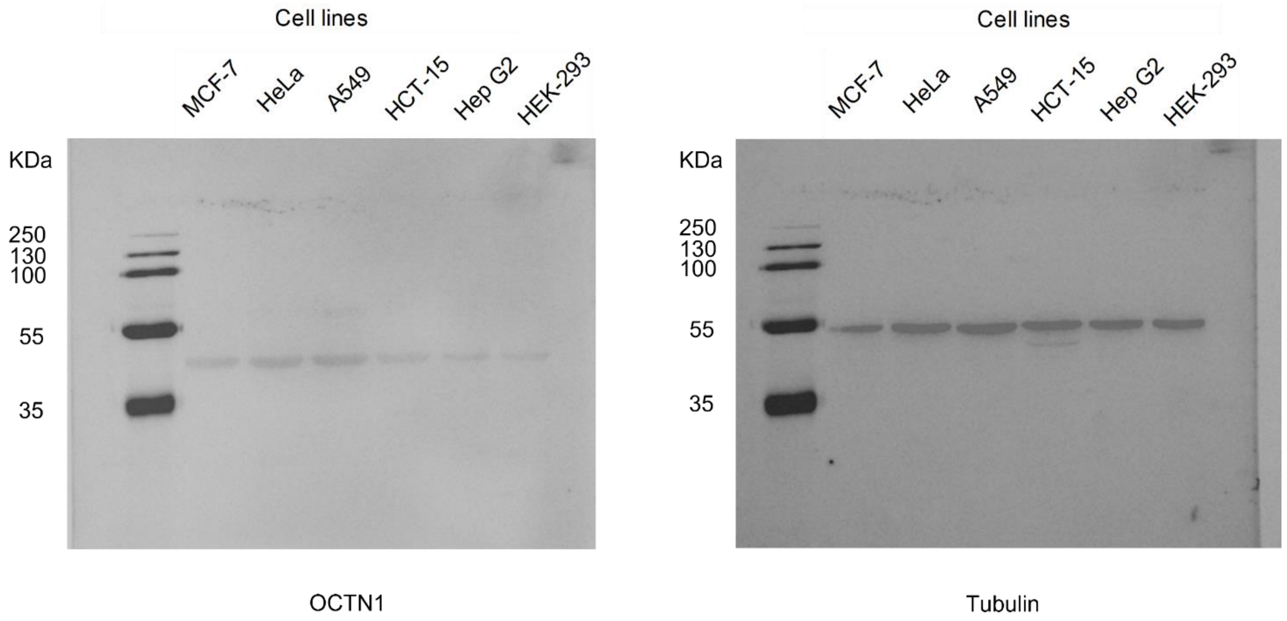

Figure 1A (Uncropped).

Cell extracts obtained as described in Section 2.2 were loaded on SDS-PAGE and blotted; OCTN1 or tubulin (loading control) was immunodetected by anti-OCTN1 or anti-tubulin, respectively. Standard markers are reported with the respective KDa.

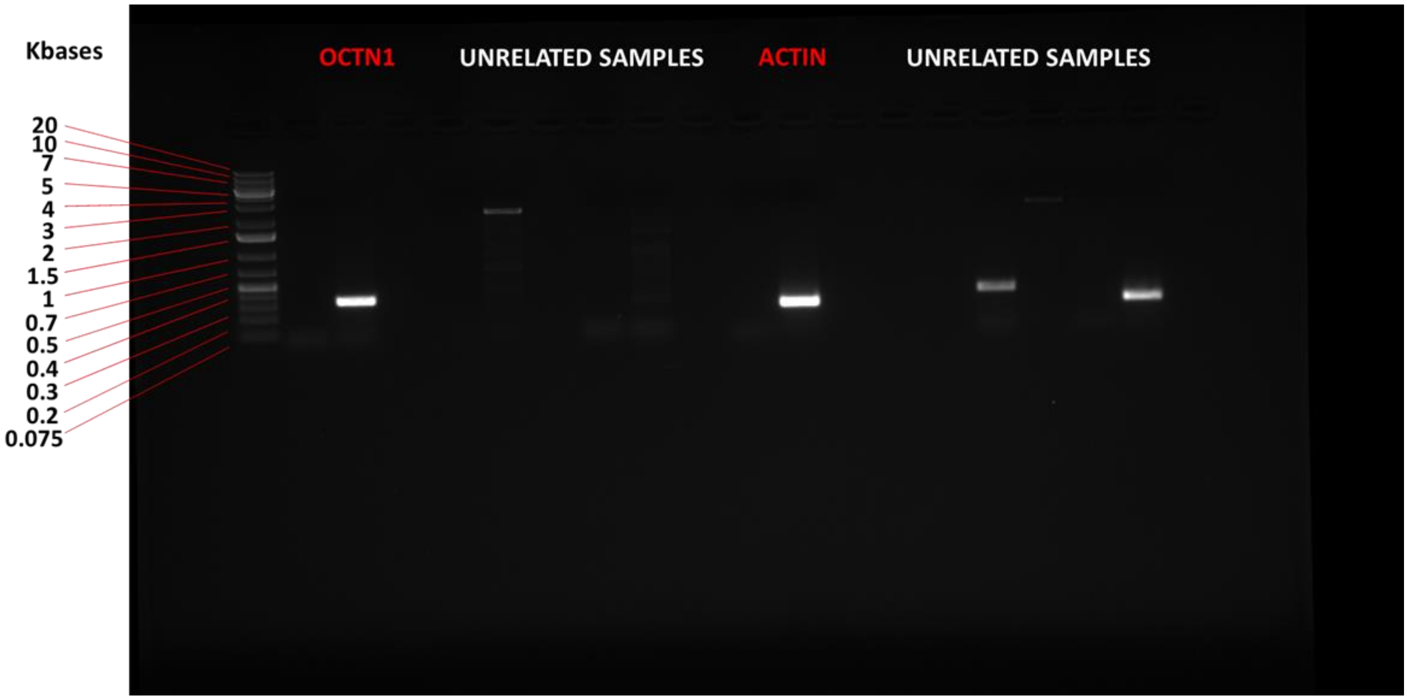

Figure 1 C (Uncropped - Identification of OCTN1 in A549 by RT-PCR.)

RT-PCR of OCTN1 and control (Actin) were performed as described in Section 2.5. Standard markers are reported in Kbases.

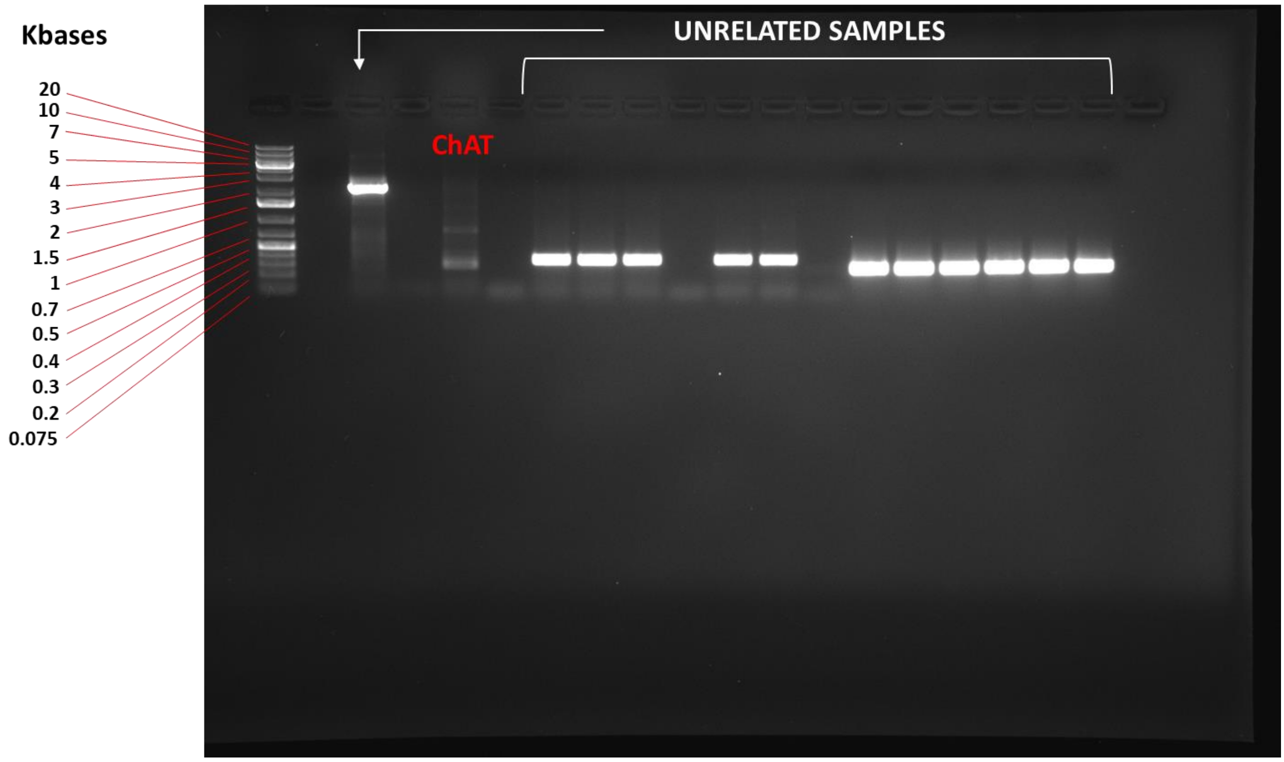

Supplementary Figure 1 (Uncropped - Identification of Choline Acetyltransferase in A549 by RT-PCR)

RT-PCR of Choline Acetyltranferase (ChAT) was performed as described in Section 2.5. Standard markers are reported in Kbases. Actin (control) is loaded in the gel of Figure 1 C.

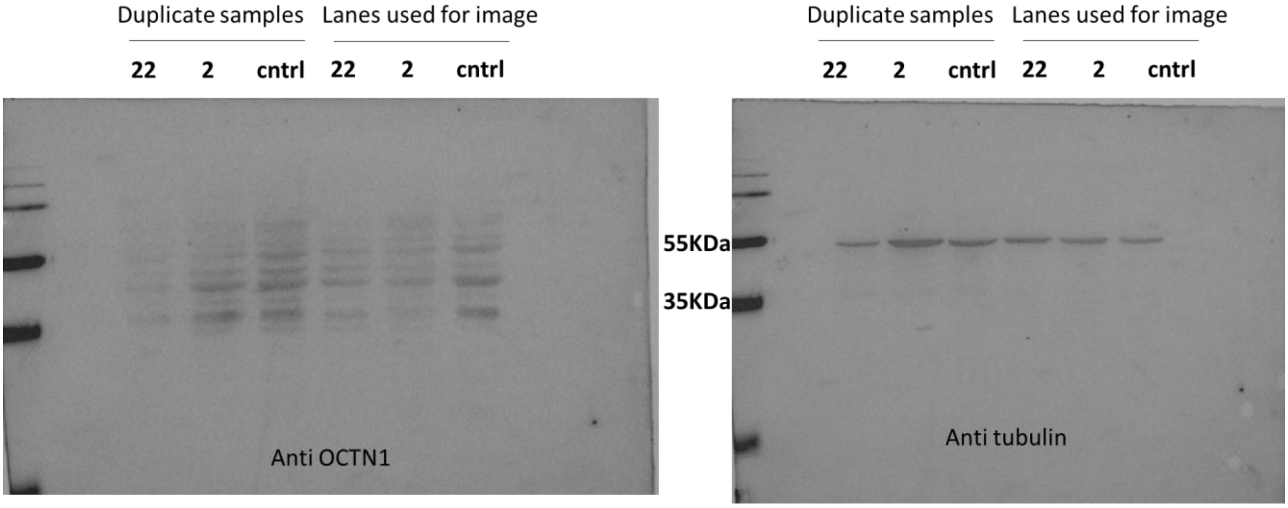

Figure 2 D (Uncropped - immunoblot analysis of Octn1-SiRNA)

Cell extracts obtained as described in Section 2.2 were loaded on SDS-PAGE and blotted; OCTN1 or tubulin (loading control) was immunodetected by anti-OCTN1 or anti-tubulin, respectively. Indicated nmol (2 or 22) of SiRNA targeting Octn1 were used with respect to control (scramble SiRNA) as described in Section 2.4.
