# Supplementary material for: OCTN1 mediates acetylcholine transport in the A549 lung cancer cells: possible pathophysiological implications
Source: Front Mol Biosci. 2024 Dec 9;11:1512530. doi: 10.3389/fmolb.2024.1512530 (PMC11666908; doi:10.3389/fmolb.2024.1512530)
Supplement: Supplementary file 2 [file Image1.pdf]

Supplementary Figure

Supplementary Figure 1

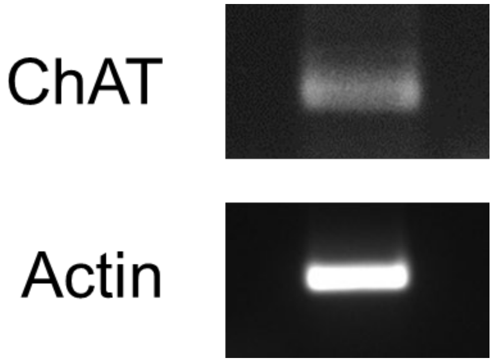

Supplementary Figure 1. Identification of Choline Acetyltransferase in A549 by RT-PCR.

RT-PCR of Choline Acetyltranferase (ChAT) and control (Actin) were performed as described in Materials and Methods
